# Prevalence, outcomes, and predictors of mortality among adult intensive care unit patients with sepsis at a Tertiary Hospital in Tanzania: A prospective cohort study

**DOI:** 10.1371/journal.pone.0351137

**Published:** 2026-06-15

**Authors:** Atala Jongo, Edwin Lugazia, Salehe Mrutu, Amina Abillah Omari, Hassani Msanga, Ansbert Sweetbert Ndebea, Felix Paul Amani

**Affiliations:** 1 Department of Anaesthesiology, Muhimbili University of Health and Allied Sciences, Ilala, Dar es Salaam, Tanzania; 2 Department of Anaesthesiology, Pwani Regional Referral Hospital-Tumbi, Kibaha-Pwani, Tanzania; Christian Medical College Vellore, INDIA

## Abstract

**Background:**

Sepsis continues to pose a significant global health challenge, particularly in low- and middle-income countries, which face a disproportionate burden of sepsis and sepsis-related deaths. The estimated prevalence of sepsis and sepsis-related mortality is higher in intensive care units than in hospitals overall. The burden can be higher in tertiary referral centers that receive patients from different regions.

This study aimed to determine the prevalence of sepsis, its outcomes, and the factors associated with these outcomes among adult patients admitted to the Intensive Care Unit (ICU) of the Muhimbili National Hospital (MNH) in Tanzania.

**Methodology:**

This prospective cohort study was conducted over a period of six months from May 16 to November 16, 2023, at MNH. A total of 248 patients were admitted during the study period and screened for sepsis on admission or for the development of sepsis during their ICU stay. Sepsis was defined according to the Sepsis-3 criteria as a suspected infection with a Sequential Organ Failure Assessment (SOFA) score ≥2 within 24 h of ICU admission. Proportions were used for descriptive statistics, and modified Poisson regression analysis was used to identify independent predictors of mortality at a 95% confidence interval, with P < 0.05 indicating statistical significance.

**Results:**

The prevalence of sepsis was 41.5%. The respiratory system was the most common source of infection (32%), and 22.3% of patients had more than one infection site. The ICU mortality rate was 55.3%, with 35% of patients developing systemic complications during their ICU stay.

Factors independently associated with mortality included multiple comorbidities (aPR 3.35, 95% confidence interval [CI], 1.20–9.32; p = 0.021) and a higher SOFA score (aPR 7.08, 95% CI 3.48–14.4; p < 0.001). Early initiation of antibiotics was independently associated with improved survival (aPR 0.47, 95% CI 0.27–0.84; p = 0.011).

**Conclusion:**

This study revealed a high prevalence of sepsis and sepsis-related mortality in the ICU. A high SOFA score and multiple complications were independent predictors of mortality. Early initiation of antibiotic therapy was an independent predictor of survival. This underscores the importance of early treatment, close monitoring, and aggressive management in patients with predictors of poor outcome.

## Background and rationale

Sepsis is defined as a life-threatening organ dysfunction resulting from dysregulated host response to infection. The disease spectrum can progress from septicemia to septic shock and multiple end-organ damage, which is associated with an increased risk of poor outcome, making early diagnosis and management crucial for a favorable outcome [1–3]. Several screening tools, such as systemic inflammatory syndrome criteria (SIRS), national early warning score (NEWS), and quick sequential organ failure assessment score(qSOFA), have been introduced for early diagnosis of sepsis and escalation of care [4,5].

Despite the availability of evidence-based guidelines such as the Surviving Sepsis Campaign (SSC), sepsis remains a major global health challenge, with an estimated 48.9 million cases and 11 million deaths annually, accounting for approximately 19.7% of all global deaths [3,6,7]. Low- and middle-income countries (LMICs) bear a disproportionate burden, accounting for approximately 85% of global sepsis cases and 84% of sepsis-related deaths. In sub-Saharan Africa (SSA), sepsis is estimated to account for approximately 17% of all adult hospital admissions and up to 31% of admissions to intensive care units. Mortality from sepsis in LMICs is higher than in high-income countries, likely reflecting differences in access to critical care, diagnostic capacity, and healthcare resources [8–10].

In Low-and-middle income countries (LMICs), patients with sepsis present with a high Burden of comorbidities compared to the non-septic patients, [11]. The respiratory tract infections represent the most common source of sepsis for out-of-hospital infections, while catheter-associated infections and ventilator-associated pneumonia are the common causes for nosocomial infections [9,12]. Increased mortality is associated with advanced age, a high SOFA score, delayed initiation of antibiotics, and vasopressor support [12–14]. Nearly a third of patients are likely to develop multiple systemic complications due to impaired organ perfusion and widespread inflammatory response during the ICU stay, which contribute to the mortality [14].

MNH is the national tertiary hospital in Tanzania, receiving patients from different regions across the country who require specialized and super-specialized care. The prevalence and outcomes of sepsis among neonates have been well studied and described; however, data on adult patients admitted to the ICU are limited [15]. Thus, this study aimed to assess the prevalence of sepsis, patient characteristics, outcomes, and factors associated with outcomes among adult patients in the ICU. This information is crucial for understanding the current burden at MNH, and understanding the clinical features and predictors of outcome will be key to risk assessment, planning, and resource allocation within the institution.

## Methodology

### Study design

This was a prospective cohort study at Muhimbili National Hospital in Dar Es Salaam, Tanzania (MNH)

### Study duration

The study was conducted for a period of six months from May 16, 2023 to November 16, 2023.

### Study area

This study was conducted at the Muhimbili University of Health and Allied Sciences (MNH) in Dar Es Salaam, Tanzania. MNH serves as a national and teaching hospital in Tanzania with a 1,500-bed capacity. The hospital receives patients from different regions across the country in need of specialized and super-specialized services across various medical specialties. The hospital has five Intensive Care Units (ICUs): Adult ICU (Medical and Surgical), Obstetric ICU, Pediatric ICU, and Neonatal ICU. The study was conducted in the adult medical and surgical ICUs, with a combined ICU bed capacity of 13 beds in the medical ICU and 11 beds in the surgical ICU. The adult medical and surgical ICU has an average admission of 300 cases per year. The hospital has 58 staff members, including four intensivists, six medical doctors, and 48 nurses.

The ICUs are equipped with facilities to provide ventilatory support, circulatory support with medications and delivery devices, intermittent hemodialysis, continuous renal replacement, and point-of-care testing (POCUS, ABG, X-ray). There is well establish protocol for the management of sepsis-induced ARDS, including proning. Currently, there is no advanced cardiac support equipment such as a ventricular Assist device or Extracorporeal Membrane Oxygenation (ECMO)

### Study population

#### Inclusion criteria.

- ICU patients aged 18 years and older who were admitted to the Medical and Surgical Intensive Care Unit during the study period.

#### Exclusion criteria.

- Patients who are readmitted to the medical and surgical intensive care unit with a prior diagnosis of sepsis within the study period.

### Sample size

The sample size was calculated using the Kish and Leslie formula. A total of 248 patients admitted to the Medical and Surgical met the inclusion criteria during the study period and were included in the study.

### Study variables

#### Dependent.

Prevalence of Sepsis and Outcome of Sepsis: Mortality vs Discharge

#### Independent.

Patient demographics, Clinical factors: SOFA score, Infection site, systemic complications, Interventions: antibiotics, vasopressors, mechanical ventilation.

### Data collection

All patients admitted to the ICU during the study period were screened for sepsis using a combination of the quick sequential organ failure assessment (qSOFA), Systemic Inflammatory Response Syndrome (SIRS) criteria, and National Early Warning scores. A suspect/confirmed infection with a SOFA score of ≥ 2, SIRS score of ≥ 2, and NEWS score of ≥ 5 was regarded as sepsis [4,5]. The SOFA score was calculated for all patients with sepsis within the first 24 h.

Additional information was collected from patients who were diagnosed with sepsis. This included demographic characteristics (age, sex, and source of admission), comorbidities, infection sites, initial treatment, and laboratory results. Additionally, the development of systemic complications, length of hospital stay, discharge, and mortality were recorded. Data were collected with the assistance of the principal investigator, aided by a trained assistant.

The infection source was determined through the patient's prior documentation, history, examination, laboratory tests, and radiological or microbiological investigations, which are part of the patient's routine care after admission to the ICU. ICU-acquired infection was defined as an infection discovered at least 48 h after ICU admission. Non-ICU-acquired infection was defined as an infection present on admission or within the first 48 h after ICU admission.

Systemic organ dysfunction and complications were based on various characteristics. Acute kidney injury (AKI) and its severity were categorized according to the Kidney Disease: Improving Global Outcomes (KDIGO) guidelines, and acute respiratory distress syndrome (ARDS) was defined using the Berlin definition. Coagulopathy was defined by platelet count, an activated partial thromboplastin time (aPPT) of 15 > 40 seconds, an INR > 1.2, or a high d-dimer above the local reference ranges. Acute liver injury was defined as evidence of hepatocellular injury (elevated AST or ALT above the upper limit of normal) in combination with clinical or biochemical features of liver dysfunction, such as jaundice or elevated bilirubin levels, in a patient without preexisting chronic liver disease. An elevated INR (≥1.5) was considered supportive of hepatic dysfunction only when accompanied by other features of liver injury and not used in isolation, given that coagulopathy in sepsis may reflect disseminated intravascular coagulation or sepsis-associated coagulopathy rather than primary hepatic failure. Myocardial infarction was determined by clinical documentation, elevated creatine kinase-MB (CK-MB) levels, or elevated troponin levels (elevations established depending on local reference ranges). Catheter-related sepsis was defined as at least one positive peripheral blood culture isolated from the catheter segment or peripheral blood. Catheter-related indicates a central line for either hemodialysis or medication administration.

### Data analysis

All questionnaires were coded and entered into IBM SPSS Statistics for Windows, version 26.0 (IBM Corp., Armonk, NY, USA). The prevalence of sepsis and clinical outcomes were expressed as percentages, and statistical diagrams and tables were used to present the data. A modified Poisson regression analysis was used to control for confounding factors and measure the strength of the association between the outcomes and associated factors. Statistical significance was set at p < 0.05, with a 95% confidence interval.

### Ethical considerations

Ethical Clearance to conduct the study was granted by the Muhimbili University of Health and Allied Sciences Institutional Review Board (IRB) Ref. No: **MUHAS-REC-03-2023-1575,** and the permission to data collection was granted by the head of clinical research, training, and consultancy at Muhimbili National Hospital (MNH), Ref. No. **MNH/CRTCU/Perm/2023/339.** Informed consent was obtained from all participants. Each participant received a detailed description of the study, and a signed consent was obtained based on their understanding and voluntary agreement to participate. Confidentiality was strictly maintained throughout the study. Identities of the participants were protected by not using their names or any identifying information. Participation was voluntary, and participants were free to withdraw from the study at any time. For critically ill patients who were unable to provide consent, a waiver was requested and approved by the ethical review board to allow the inclusion in the study, and permitted the researcher to review relevant patient information after the study proposal was submitted and approved.

## Results and discussion

### Results

#### Prevalence of sepsis.

**Fig 1:**
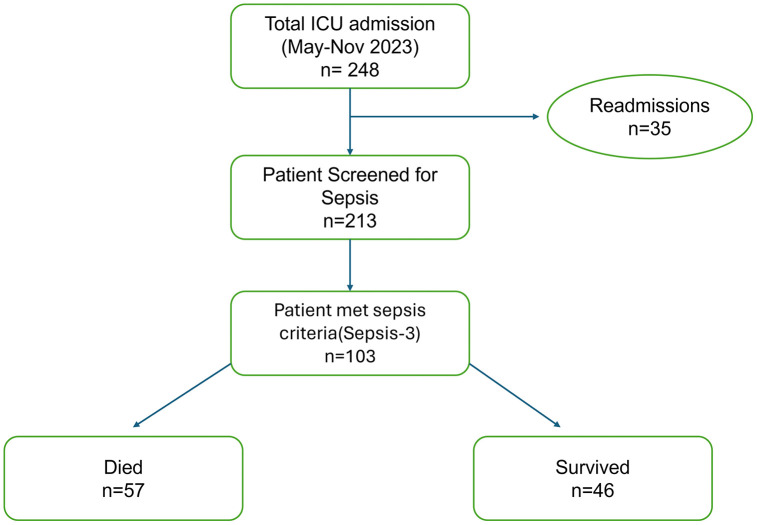
Participant flow chart of adult Intensive Care Unit patients included in the study.

A total of 248 patients were admitted to the ICUs (Medical and Surgical) during the study, which was higher than the expected number of admissions based on previous data. This may reflect seasonal variation or improved access to ICU service (Fig 1).

The prevalence of sepsis was 41.5% (103/248). Among patients with sepsis, 90 (87.4%) were identified on admission (early-onset), while 13 (12.6%) developed sepsis during ICU stay (ICU-acquired). Among patients with sepsis, the majority (62.1%) were men, 48.5% were over the age of 50, and hypertension was the most common comorbidity among these patients. Among these patients, the majority (50.5%) were diagnosed at the Emergency Department, with 12.6% developed sepsis in the course of ICU stay. The majority of patients (71.8%) with sepsis were recorded in the medical ICU. At least one comorbidity was found in 80.6% of patients, with hypertension (27.2%) being the most frequent, and 15.5% had more than one comorbidity. The common site of infection was the respiratory system (32%), and about 22.3% had more than one infection site. (Table 1).

**Table 1:** Demographic characteristics of patients admitted to the ICU with sepsis at MNH,2023.

Variable	Response	Frequency(n = 103)	Percentage
Age	18-25	11	10.7
	26-30	13	12.6
	31-40	15	14.6
	41-50	14	13.6
	>50	50	48.5
Sex	Male	64	62.1
	Female	39	37.9
Source of Admission	Emergency Department	52	50.5
	Ward	38	36.9
	ICU	13	12.6
Intensive care Unit	Medical	74	71.8
	Surgical	29	28.2
Source of Infection	Blood	16	15.5
	Respiratory	33	32
	Urinary Tract	10	9.7
	Intrabdominal	6	5.8
	Surgical Site	12	11.7
	Multiple sites	23	22.3
	Undetermined	3	2.9
Comorbidities	Hypertension	28	27.2
	Diabetes	11	10.7
	Congestive Heart Failure	5	4.9
	Kidney disease	15	14.6
	Liver disease	4	3.9
	Leukemia	2	1.9
	HIV/AIDS	2	1.9
	Multiple Comorbidities	16	15.5
	None	20	19.4

#### Clinical outcomes and complications among patients with sepsis.

Among the 103 patients with sepsis, 55.3% died and 44.7% were discharged after treatment. During the ICU stay, 35% of the patients developed systemic complications, with acute renal failure (36.1%) being the most common, followed by acute respiratory distress syndrome (ARDS) (16.8%). Multiple systemic complications were observed in 19.4% of the patients. The median length of ICU stay was 8 days (IQR 5–12) among patients who died, and the mean length of stay was 10 ± 8.2 days. (Table 2).

**Table 2:** Outcomes and systemic complications of patients admitted to ICU with sepsis admitted to the ICU at MNH, 2023.

Variable	Response	Frequency	Percentage
Outcome	Died	57	55.3
	Discharged	46	44.7
Systemic Complications	Acute kidney injury	13	36.1
	Coagulopathy	4	11.1
	Liver Failure	3	8.3
	ARDS	6	16.8
	Myocardial Ischemia	3	8.3
	Multiple System (>1)	7	19.4
	Number of Patients	Median LOS, days (IQR)	Mean LOS ± SD (days)
Died from Sepsis	57	8(5-12)	10.0 ± 8.2
Survived Sepsis	46	10(6-16.8)	11.6 ± 6.5

#### Factors associated with outcomes.

In a univariate modified Poisson analysis showed those aged 26–30 years had a lower crude prevalence of death (cPR 0.63, 95% CI 0.41–0.98; p = 0.04), patients with multiple comorbidities (cPR 1.61, 95% CI 1.17–2.2; p = 0.004), Multiple sources of infections (cPR 1.52, 95% CI 1.10–2.10; p = 0.011), systemic complications (cPR 1.96, 95% CI 1.61–2.39; p < 0.001), multiple complications (cPR 1.96, 95% CI 1.61–2.39; p = 0.004) and higher SOFA Score were statistically significant associated with higher mortality (cPR 7.14, 95% CI 3.58–14.24 p < 0.001). Early initiation of antibiotics was statistically significantly associated with survival (cPR 0.32, 95% CI 0.21–0.51; p < 0.001).

In the multivariate analysis presence of multiple comorbidities (aPR 3.35, 95% CI 1.20–9.32; p = 0.021), a higher SOFA score (SOFA ≥15 (aPR 7.08, 95% CI 3.48–14.40; p < 0.001)) were independent predictor of high mortality, and early initiation of antibiotics was an independent predictor of survival (aPR 0.47, 95% CI 0.27–0.84; p = 0.011). Additionally, patients aged 26–30 years had a lower adjusted prevalence of death (aPR 0.58, 95% CI 0.34–0.98; p = 0.04). (Table 3)

**Table 3:** Multivariate modified Poisson regression analysis on factors associated with ICU mortality among patients with sepsis at MNH,2023.

Variable	Response	Outcome		cPR(95% CI)	p-Value	aPR(95% CI)	p-Value
		Died	Survived				
Age	18-25	4(36.4%)	7(63.6%)	0.82 (0.55–1.23)	0.34	1.00 (0.48–2.08)	1
	26-30	6(46.2%)	7(53.8%)	0.63 (0.41–0.98)	0.04	0.58 (0.34–0.98)	0.04
	31-40	8(53.3%)	7(46.7%)	0.73 (0.49–1.08)	0.12	0.78 (0.42–1.46)	0.45
	41-50	8(57.1%)	6(42.9%)	0.85 (0.56–1.29)	0.45	1.37(0.55-3.41)	0.5
	>50	31(62%)	19(38%)	1		1	
Sex	Male	35(54.7%)	29(45.3%)	1.03 (0.72–1.47)	0.86	–	–
	Female	22(56.4%)	17(43.6%)	1		1	
Source of Admission	EMD	29(55.8%)	23(44.2%)	1		1	
	General Ward	23(60.5%)	15(39.5%)	1.08(0.77-1.51)	0.65	1.1(0.79-1.54)	0.56
	ICU	5(38.5%)	8(61.5%)	0.69(0.39-1.22)	0.2	0.74(0.44-1.25)	0.26
Multiple Sources of Infection	Yes	18(75%)	6(25%)	1.52(1.1-2.1)	0.011	1.02(0.85-1.23)	0.81
	No	39(49.4%)	40(50.6%)	1		1	
Comorbidity	Present	50(62.5%)	30(37.5%)	0.90 (0.52–1.56)	0.7	–	–
	Absent	7(30.4%)	16(69.6%)	1			
Antibiotic within 1hour	Yes	15(27.8%)	39(72.2%)	0.32 (0.21–0.51)	<0.001	0.47 (0.27–0.84)	0.011
	No	42(85.7%)	7(14.3%)	1		1	
SOFA Score	≤10	7(14%)	43(86%)	1		1	
	11-14	23(88.5%)	3(11.5%)	6.32(3.14–12.71)	<0.001	6.25 (3.10–12.58)	<0.001
	≥15	27(100%)	0(0%)	7.14(3.58–14.24)	<0.001	7.08 (3.48–14.40)	<0.001
Systemic Complication	Present	26(72.2%)	10(27.8%)	1.96 (1.61–2.39)	<0.001	0.87 (0.40–1.88)	0.72
	Absent	31(46.3%)	36(53.7%)	1		1	
Multiple Complications (>1)	Present	9(100%)	0(0%)	1.96 (1.61–2.39)	0.004	1.08 (0.88–1.33)	0.46
	Absent	48(51.1%)	46(48.9%)	1		1	
Multiple Comorbidities (>1)	Present	13(81.3%)	3(18.8%)	1.61 (1.17–2.20)	0.003	3.35 (1.20–9.32)	0.021
	Absent	44(50.6%)	43(49.4%)	1			
Need for Vasopressors	Yes	34 (58.6)	24 (41.4)	1.03 (0.72–1.46)	0.889	–	–
	No	23 (51.1)	22 (48.9)	1		1	
Mechanical Ventilation	Yes	36 (56.3)	28 (43.7)	0.87 (0.62–1.23)	0.441	–	–
	No	21 (53.8)	18 (46.2)	1		1	

*cPR = Crude Prevalence Ratio aPR: Adjusted Prevalence Ratio.

## Discussion

### Prevalence of sepsis

The study revealed a high burden of sepsis (41.5%) among adult ICU-admitted patients admitted to the ICU. This finding is substantially higher than that of high-income countries but is consistent with the conclusions of most LMICs, including sub-Saharan countries, despite being slightly higher than the overall pooled ICU sepsis prevalence in SSA (31% (95% CI: 24–38%)) [9,10,13,16–18]. This has been attributed to the high burden of infectious diseases in the region, delayed presentation and detection, and constraints of the healthcare system. Similar findings have been reported in neighboring East African countries, with other studies reporting an ICU prevalence of up to 61.3% [19]. The variations depend on the case definitions, case mix, number of patients, and recruitment methods(prospective/retrospective). Overall, sepsis contributes to significant ICU admissions in this region.

The majority of patients with sepsis were > 50 years of age, similar to other studies conducted in a tertiary hospital's ICUs, which showed that patients with sepsis in the ICU tended to be older. However, a few studies in the region have reported younger patients, reflecting the combined burden of infectious diseases. Additionally, most patients with sepsis were male, and most had at least one comorbidity, with hypertension being the most common [11,13,20]. The respiratory system was the most common infection site in this study. Similarly, a high burden of comorbidities among patients with sepsis, with the respiratory system being the most common source of infection, has been observed [12,20,21]. This shows the similarity in patient profiles suffering from sepsis across the region. Pre-existing comorbidities usually alter patients’ immunity and make them susceptible to infections. The interaction between mechanical ventilation, impairment of immunity, and physiological vulnerability of the respiratory tract among critically ill patients accounts for the respiratory system being the most common source of infection.

### Outcomes among adult patients with sepsis in the ICU

More than half of the mortality was recorded among patients admitted to the ICU with sepsis. This was significantly higher than in most high-income settings but consistent with outcomes reported across sub-Saharan Africa. This can be attributed to delayed presentation, limited access to early critical care, and a constrained referral hospital, which leads to a high severity of illness at admission to a tertiary facility [12,14,19,13,22,23].

Nearly one-third of patients develop systemic complications following sepsis diagnosis during their ICU stays. Acute renal failure and acute respiratory distress syndrome were the most common complications, and one-fifth of patients had more than one systemic complication. These findings align with the available literature, which has shown that the development of system dysfunction fits in the sepsis spectrum, with acute renal injury and acute respiratory failure being the most prevalent conditions in the ICU [22,24]. Organ dysfunction develops as a result of sepsis-induced hypoperfusion and dysregulation of microcirculation, with the renal and respiratory systems being the most vulnerable systems. A subset of patients develops multiple complications that substantially increase their mortality risk [24].

The median (8days (IQR5–12) and mean(10 ± 8.2days) ICU length of stay for non-survivors was longer compared to most other countries in the SSA region(3–5days). The picture in most of Sub-Saharan Africa is a short ICU length of stay for non-survivors [22,25]. This can be due to differences in the severity of clinical presentations of patients on admission to the ICU and early care following admission to the ICU. The longer ICU stay in our study can be due to patient deterioration over the course of the ICU stay after initial stabilization. The MNH ICUs are well equipped with facilities for early resuscitation and stabilization, such as mechanical ventilators, vasopressors, oxygen supply, antibiotics, and a clear pathway for surgical source control, which offers an early survival advantage compared to other SSA settings where there are scarce resources [26]. However, due to a lack of advanced organ support interventions such as VA and ECMO, patient deterioration to multiorgan dysfunction in the course of ICU stay can limit the chances of survival following initial stabilization. The length of stay for survival was longer than for non-survival, which mirrors other studies [27].

### Predictors of outcomes among adult patients with sepsis in the ICU

The study shows that having multiple sources of infections, multiple comorbidities, systemic complications, and a high SOFA score were statistically significantly associated with poor outcome, while early initiation of antibiotics was protective against mortality. The findings from this study align with the available literature. Multiple infection sites and comorbidities predispose the patient to develop severe illness, while a high SOFA score and the development of systemic complications are signs of severe illness with increased risk of mortality [9,12,14,28]. Early initiation of antibiotics allows rapidly elimination of microbial organisms that will prevent further injury, progression of organ failure, and death. However, the protective role of antibiotics should be interpreted cautiously, as the severity of illness has an impact on outcome. Although younger age is generally associated with improved outcomes in sepsis, our findings suggest a relatively uniform mortality risk across age groups after adjustment. While the 26–30-year age group demonstrated a lower adjusted prevalence ratio, this pattern was not consistent across other age categories, and confidence intervals were wide. Notably, the mortality risk among patients aged 18–25 years was comparable to that of patients older than 50 years. This suggests that, in our setting, the severity of illness at presentation may outweigh the protective effect of younger age. Possible explanations include delayed presentation leading to advanced disease, selective ICU admission of only the most critically ill younger patients, and the dominant influence of organ dysfunction, as reflected by SOFA scores, on outcomes. These findings highlight that in resource-limited settings, mortality may be driven more by disease severity than by demographic factors such as age.

In multivariate analysis, having multiple comorbidities and a higher SOFA score remained independent predictors of mortality, while young age and early initiation of antibiotics were independent predictors of survival. This finding aligns with the existing literature, both regionally and globally [14,29–31]. Multiple infection sites and systemic complications lose their statistical significance after adjustment, which shows their contributions in the position along the pathway of severe sepsis to death, but might not have a direct influence on mortality. Another observation was that a patient with multiple systemic complications and a SOFA score of more than 14 had 100% mortality, which shows their significance in mortality, as both of them indicate a severe form of the disease.

## Limitations

- The study assessed patients over a six-period time, which might influence the research findings due to periodic variation.
- In our study, we didn’t assess the care provisions that might have provided an insight on better understanding of the study outcomes.

## Conclusion

The study revealed a high prevalence of sepsis and case-related mortality, which reflected the picture and clinical profile of sub-Saharan African settings. Early initiation of antibiotics has been shown to impact survival, underscoring the importance of early recognition and treatment of sepsis. Multiple infection sites, multiple comorbidities, systemic complications, and a high SOFA score were associated with mortality, whereas multiple comorbidities and a high SOFA score were independent predictors of mortality. This emphasizes the importance of early prevention of progression to systemic complications and close monitoring and aggressive management in patients with high SOFA scores, multiple comorbidities, multiple infection sites, and systemic complications.

Since this study found high mortality in patients with sepsis, we propose a study that will examine the level of adherence to sepsis guidelines in our setting, which has been shown to improve outcomes.
