# Supplementary material for: Prevalence, outcomes, and predictors of mortality among adult intensive care unit patients with sepsis at a Tertiary Hospital in Tanzania: A prospective cohort study
Source: PLoS One. 2026 Jun 15;21(6):e0351137. doi: 10.1371/journal.pone.0351137 (PMC13268162; doi:10.1371/journal.pone.0351137)
Supplement: Attachment — Submitted filename: Review for manuscript PONE-D-26-06135.docx [file pone.0351137.s001.docx]

**Peer Review**

Manuscript: PONE-D-26-06135

*Prevalence of Sepsis and Outcome of Adult Patients Admitted to the Intensive Care Unit at a Tertiary Teaching Hospital in Tanzania: A Prospective Cohort Study*

**1. Summary**

This manuscript reports a prospective observational study conducted in adult medical and surgical ICUs at Muhimbili National Hospital, Tanzania (May–November 2023). Among 248 ICU admissions, 103 patients met the authors' sepsis criteria, yielding a reported prevalence of 41.5% and an ICU mortality rate of 55.3%. The study further examines predictors of mortality, identifying higher SOFA scores, multiple comorbidities, and delayed antibiotic administration as key contributing factors.

The research topic is clinically significant given the substantial burden of sepsis in low- and middle-income countries (LMICs) and sub-Saharan Africa (SSA). However, major methodological and reporting concerns—particularly the sepsis case definition, internal inconsistencies in statistical methods, and high risk of time-related and severity confounding in the antibiotic timing analysis—currently preclude reliable interpretation of the findings.

**2. Major Comments**

**1. Sepsis case definition is not aligned with Sepsis-3 and conflates screening tools with diagnostic criteria**

The manuscript invokes the Sepsis-3 framework conceptually, yet the Methods section defines sepsis as qSOFA ≥ 2 and/or SIRS ≥ 2 and/or NEWS ≥ 5. These are screening or triage tools, not diagnostic definitions; their use is likely to produce substantial misclassification and poor comparability with the broader literature.

**Required revision:** Clearly distinguish between “suspected/possible sepsis” and “Sepsis-3–defined sepsis.” Where feasible, provide a sensitivity analysis using suspected infection plus SOFA increase ≥ 2 (or SOFA ≥ 2 within 24 hours), and report how prevalence estimates and effect measures change accordingly.

**2. Study design is mislabeled as “cross-sectional” despite time-to-outcome follow-up**

Patients were followed through ICU discharge or death, and the analysis examines longitudinal predictors of outcome—features consistent with a prospective cohort design rather than a true cross-sectional study.

**Required revision:** Revise the study design designation throughout the manuscript (title, Methods, and Abstract) and ensure that STROBE reporting elements align with cohort study standards.

**3. Statistical methods are internally inconsistent and may be incorrectly implemented or interpreted**

The Abstract and Results sections report modified Poisson regression with prevalence ratios (cPR and aPR), whereas the Methods section describes logistic regression. Furthermore, the multivariable results for SOFA score categories appear inconsistent with the observed crude mortality patterns—for example, a reported 100% mortality in the SOFA > 14 group is difficult to reconcile with adjusted PRs at or below 1.0 for certain categories.

**Required revision:** Align the statistical methods described in the Methods with those actually used in the Analysis. Provide a clear and internally consistent presentation of regression results, and verify that the direction and magnitude of adjusted estimates are analytically coherent.

**3. Minor Comments**

1. Provide a participant flow diagram illustrating the progression from total ICU admissions through exclusions and readmissions, to sepsis identified on admission versus ICU-acquired, and through to final outcomes.
2. Report missing data explicitly for key variables, including SOFA component scores, laboratory values, infection site determination, and antibiotic administration timing.
3. Clarify whether the “ICU-acquired infection” subgroup (onset ≥ 48 hours after admission) differs from early-onset cases with respect to causative pathogens and clinical outcomes. At minimum, present stratified descriptive results for this subgroup.

**4. Recommendation**

**Decision: Major Revision**

This manuscript addresses an important public health question with valuable ICU-level data from Tanzania. Nonetheless, the current version contains fundamental definitional and analytic problems—particularly regarding the sepsis case definition, potential antibiotic timing bias, and internal statistical inconsistencies—that must be resolved before the results can be interpreted with confidence. The authors are encouraged to address all major comments thoroughly and to revisit the study design classification, which appears inconsistent with the longitudinal nature of the data.
