# Supplementary material for: Prevalence, outcomes, and predictors of mortality among adult intensive care unit patients with sepsis at a Tertiary Hospital in Tanzania: A prospective cohort study
Source: PLoS One. 2026 Jun 15;21(6):e0351137. doi: 10.1371/journal.pone.0351137 (PMC13268162; doi:10.1371/journal.pone.0351137)
Supplement: Attachment — Submitted filename: sepsis in tanzania review.docx [file pone.0351137.s002.docx]

Thank you for the opportunity to review “Prevalence of sepsis and outcome of adult patients admitted in an intensive care unit patients in a tertiary teaching hospital in Tanzania: A prospective cross-sectional Study”, which, as the title states, is a cross-sectional study of patients with sepsis at a single tertiary center in the nation of Tanzania. While the results of single-center cross sectional studies such as these are not usually practice-changing, they provide a profile of disease prevalence and characteristics in a particular geographic region which is relevant to the medical literature, particularly in lower and middle-income countries (LMICs) where resource availability may be different than that in the resource rich countries which produce most of the medical literature.

In terms of novelty, I do not find other papers addressing sepsis incidence and outcomes among adult ICU patients in the nation of Tanzania, though I did note this similar paper which examined sepsis outcomes among adult emergency department patients with sepsis in Tanzania (Bonnewell et al, Open Forum Infect Dis, 2024, PMID: 39877396).

Publication is possible, but significant changes to the analysis and conclusions would be needed.

**Major concerns:**

1. The criteria the authors have used to identify sepsis (qSOFA 2 or more, SIRS 2 or more, or NEWS 5 or more) are not entirely robust. Since the sepsis-3 criteria were published (Singer JAMA 2016), the use of qSOFA as a screening tool has fallen out of favor and is no longer recommended in the 2021 surviving sepsis guidelines. SIRS and NEWS may be used as screening tools, but an established diagnosis of sepsis is made by a SOFA score increase of 2 or more from baseline in concert with a known or suspected infection. The authors state that they calculated SOFA scores within 24 hours of ICU admission for all patients; using this as the basis for sepsis diagnoses would be more reliable than the screening tools, and results would be more readily comparable with results from other studies.
2. One of the authors’ main conclusions is that their observed ICU sepsis mortality rate of 55% is much higher than that observed in resource-rich countries. While resource availability is undoubtedly linked to survival rates, the degree of discrepancy should be further explored. I recommend one of two approaches. First, the authors could provide literature citations of mortality rates among ***ICU-admitted*** patients with sepsis in resource rich countries (the citations the authors provide all seem to apply to LMICs). Alternatively, the authors could state the number of septic patients in their study who also qualify as having septic shock (Shankar-Hari, JAMA, 2016, PMID: 26903336). This would give a better idea of how sick the cohort is and whether the expected mortality rate would be closer to the 10% seen in sepsis alone (Singer, JAMA, 2016) or the 42% expected for septic shock (Shankar-Hari).
3. The motivation for and application of the univariate regression analysis needs to be clearer. Use of the multivariable logistic regression analysis makes sense, but what was the univariable regression meant to show that couldn’t be determined from the other single variable analyses utilized by the authors (chi-squared and fisher exact testing)? It adds clutter to the results without significantly changing the conclusions; I would recommend removing the univariable regression analysis unless there was a specific purpose for which the authors have included it (if so, this should be stated).
4. The authors conclude that younger age is associated with improved survival because of a statistically significant decrease in sepsis mortality in the 26-30 year old age bracket, and such a conclusion would align with widely accepted prior research. However, the distribution of mortality risk, when taken as a whole, actually shows a relatively flat risk of sepsis mortality relative to age, particularly when adjusted odds ratios are considered (table 4). That the adjusted odds ratio of mortality for the 18-25 age group is identical to the >50 age group is particularly striking. Rather than trying to sweep over this the authors should lean into it and further explore in their discussion what could be driving it. To me this is the most interesting finding of the paper.

**Minor concerns:**

**Introduction:**

1. In the second paragraph the authors note several statistics as percentages but it’s unclear what the percentages refer to. “…with approximately 85% of all cases and 84% of all sepsis-related deaths”: is this saying 85% of all ICU admissions are patients with sepsis and 84% of all hospital deaths are sepsis related? This needs to be more clear. Similarly “…17% of all adult hospital admissions and 31% of ICU admissions” what are the 17% and 31% referring to?
2. In the third paragraph of the introduction (“Patients with sepsis…”), the authors should state the specific population they are referring to here. Are these statements about sepsis trends worldwide? Sepsis trends specific to LMICs? Or specific to a particular geographic region? Based on the citations used it appears to be specific to LMICs or particular parts of Africa, but that should be stated in the text.
3. In the fourth paragraph of the introduction, what are “under-fives”? This needs to be defined in the text.

**Methods:**

1. In study duration, I recommend writing out the dates of the study longhand (e.g. May 16, 2023) to avoid international differences related to ordering day and month in shortform.
2. Per the methods section, the MICU/SICU at the study site admits about 300 patients per year, but in 6 months of sampling there were 248 patients admitted and included in the study (suggesting a typical annual volume of more like 500 patients per year). There needs to be some explanation about how these numbers are coherent with one another.
3. More description of the capabilities of the study center would be helpful. Is continuous renal replacement therapy available? What about hemodialysis more generally? VA- or VV-ECMO? For subjects with severe sepsis-induced ARDS is there a protocol for prone positioning? Inhaled prostacyclins (e.g. velitri or flolan)?
4. An INR of >1.5 was utilized to establish a diagnosis of liver dysfunction, but among septic patients and elevated INR could also be caused by disseminated intravascular coagulopathy (DIC) or simply sepsis-associated coagulopathy.
5. The authors should state why in their singe variable analysis they sometimes used chi-squared testing and sometimes used Fisher Exact testing. It appears they are appropriately using Fisher Exact testing for variables with low frequency outcomes, but this should be explicitly stated.

**Results**

1. In Table 3, p-values should not be expressed as “0”, but rather as an upper boundary (e.g. “P < 0.0001”).
2. [Related to major concern 3 above]: Table 4 is not very clear. What do the acronyms cPR and aPR refer to? I think these are some type of crude and adjusted odds ratios from the regression analyses but the acronyms should be defined. Additionally, the cPR and aPR column titles should be changed to match the format in which they are reported in the table (ie. “cPR (95% CI)”). Finally, if the univariate regression is kept (see major concern 3) the authors should be very deliberate about which regression analysis they are referring to. In the paragraph starting with “In a univariate modified Poisson analysis…” it appears both cPRs and aPRs are referenced without clear distinction between univariate and multivariate analysis models.

**Discussion:**

1. The authors note that their LOS among non-survivors was longer than that for non-survivors elsewhere in Sub Saharan Africa (SSA) and attribute this a slow decline after an initial period of stabilization. The important question raised by this, though, is why the patients in the study center were able to achieve the initial period of stabilization whereas this was not achieved at other sites in SSA. Does this have to do with the way resources are allocated, or something else? The authors should comment on this.

**Typos**:

Overall the manuscript reads well but the authors should again proofread their manuscript for typos. A few that I found:

1. The word "patient" appears duplicated in the title. I recommend modification to "Prevalence of Sepsis and Outcome among Adult Intensive Care Unit Patients in a Tertiary Teaching Hospital in Tanzania: A Prospective Cross-Sectional Study"

2. In the abstract background paragraph, I believe the authors intend "...particularly in low- and middle-income countries, which face a disproportionate burden...”

3. Last sentence of the abstract results paragraph should read "were independent predictors of high mortality"
4. At the top of the page containing Table 4, I believe the authors intend “were independent predictors of high mortality, and early initiation of antibiotics…”
5. In the discussion section titled Outcomes among adult patients with sepsis in the ICU, first paragraph, I believe the authors intend: “Early initiation of antibiotics allows rapid elimination of…”
