# Supplementary material for: Prevalence, outcomes, and predictors of mortality among adult intensive care unit patients with sepsis at a Tertiary Hospital in Tanzania: A prospective cohort study
Source: PLoS One. 2026 Jun 15;21(6):e0351137. doi: 10.1371/journal.pone.0351137 (PMC13268162; doi:10.1371/journal.pone.0351137)
Supplement: Attachment — Submitted filename: Response to Reviewers.docx [file pone.0351137.s004.docx]

# Response to Reviewers

Manuscript ID: PONE-D-26-06135

Title: Prevalence, Outcomes, and Predictors of Mortality Among Adult Intensive Care Unit Patients with Sepsis at a Tertiary Hospital in Tanzania: A Prospective Cohort Study

Dear Editor and Reviewers,

We sincerely thank you for your thorough and insightful review of our manuscript. We appreciate the constructive comments, which have significantly improved the clarity, methodological rigor, and overall quality of our work. We have carefully addressed all comments and revised the manuscript accordingly. Below is a detailed, point-by-point response.

**REVIEWER 1 – MAJOR COMMENTS**

1. Sepsis definition not aligned with Sepsis-3

Response:

We agree with this important observation. The manuscript has been revised to clearly distinguish between screening and diagnostic criteria. All cases were included in the study after evidence of infection. This was included in the previous submission on the description of sources of infection.

Where feasible, we have clarified the implications of this definition and improved comparability with existing literature.

2. Study design mislabeled as cross-sectional

Response:

We agree and have corrected this throughout the manuscript. The study is now consistently described as a prospective cohort study, as patients were followed from ICU admission until discharge or death. The title, abstract, and methods have been revised accordingly.

3. Statistical inconsistencies (Poisson vs logistic regression, SOFA findings)

Response:

We thank the reviewer for highlighting this inconsistency. The statistical methods have been fully revised for clarity and consistency. The manuscript now exclusively reports modified Poisson regression for estimation of adjusted prevalence ratios (aPR). The regression models have been re-evaluated, and SOFA categories were restructured to ensure logical consistency with observed mortality patterns.

**REVIEWER 2 – MAJOR COMMENTS**

1. Sepsis criteria robustness

Response:

We have revised the sepsis definition to align with Sepsis-3 criteria and clarified the role of screening tools. This improves diagnostic accuracy and comparability with global literature.

2. High mortality compared to resource-rich settings

Response:

We have added the literature on the HIC as advised.

3. Univariate regression analysis redundancy

Response:

In the revised analysis, we have removed the Chi-square and Fisher's exact testing and left the univariate analysis, which provided the strength and level of significance. Those variables with a p-value < 0.2 were included in the multivariable analysis regression model to control for confounding.

4. Age and mortality interpretation

Response:

We appreciate this insightful observation. We have revised the interpretation to reflect that mortality risk was relatively uniform across age groups after adjustment.

Although one subgroup (26–30 years) showed statistical significance, this was not consistent across other groups, and confidence intervals were wide. We now interpret this finding as suggesting that illness severity, rather than age alone, is the dominant determinant of outcome.

MINOR COMMENTS

1. Introduction, unclear percentages

Response:

We have revised the text to explicitly define all percentages. The denominators (global cases, global deaths, hospital admissions, ICU admissions) are now clearly specified.

2. Population clarification (LMIC vs global vs regional)

Response:

We have clarified that the referenced trends relate specifically to LMICs and sub-Saharan Africa.

3. Definition of “under-fives.”

Response:

The exact term and age group studied have been revised.

4. Study duration format

Response:

Dates are now written in full format (e.g., May 16, 2023).

5. ICU admission volume discrepancy

Response:

We have clarified that increased admissions during the study period likely reflect referral patterns and variability in patient flow.

6. ICU capability description

Response:

We have expanded the Methods section to describe ICU resources, including availability of mechanical ventilation, vasopressors, intermittent hemodialysis, CRRT, and absence of ECMO.

7. INR and liver dysfunction (DIC concern)

Response:

We revised the definition of acute liver injury to avoid reliance on INR alone and clarified that coagulopathy in sepsis may reflect DIC rather than hepatic dysfunction.

8. P-values reported as 0

Response:

All such values have been corrected to “p < 0.001”.

9. Table clarity (cPR/aPR definitions)

Response:

The cPR and aPR acronyms have been defined. An appropriate description of cPR and aPR has been revised in the results section.

10. ICU length of stay explanation.

Response:

We have expanded the discussion to explain longer LOS among non-survivors in our setting, highlighting differences in early stabilization, resource availability, and disease trajectory.

11. Typographical errors

Response:

All typographical and grammatical issues have been corrected as suggested.

**ADDITIONAL REVISIONS (EDITOR COMMENTS)**

Participant flow diagram

Response:

A flow diagram has been added showing ICU admissions, sepsis identification, subgroup classification, and outcomes.

Missing data reporting

Response:

In our study, we had 3 patients whose source of infections undocumented, which we labeled as undetermined in the source of infection in Table 1

ICU-acquired vs early-onset sepsis

Response:

We have added a stratified descriptive comparison of early-onset and ICU-acquired sepsis, including clinical characteristics and outcomes.

**CONCLUSION**

We believe that these revisions have addressed all major and minor concerns raised by the reviewers and have substantially strengthened the manuscript. We are grateful for the constructive feedback and hope the revised version meets the journal’s standards for publication.

Sincerely,

Atala Jongo

On behalf of all authors
