# Supplementary material for: Prevalence, outcomes, and predictors of mortality among adult intensive care unit patients with sepsis at a Tertiary Hospital in Tanzania: A prospective cohort study
Source: PLoS One. 2026 Jun 15;21(6):e0351137. doi: 10.1371/journal.pone.0351137 (PMC13268162; doi:10.1371/journal.pone.0351137)
Supplement: Peer review history

# Peer review: Decision Letter 0

**DOI:** 10.1371/journal.pone.0351137.r001

**Authors:** Ramya Iyadurai

21 Mar 2026

PONE-D-26-06135

Prevalence of sepsis and outcome of adult patients admitted in Intensive care unit patients in a tertiary teaching hospital in Tanzania: A Prospective Cross-Sectional Study

PLOS One

Dear Dr. JONGO,

Thank you for submitting your manuscript to PLOS ONE. After careful consideration, we feel that it has merit but does not fully meet PLOS ONE’s publication criteria as it currently stands. Therefore, we invite you to submit a revised version of the manuscript that addresses the points raised during the review process.

Dear Authors thank you for an interesting study in a area of critical need. , kindly address  the following concerns;

Add a report on missing data Kindly ensure that your statistical methods is synchronous with the description of statistical methods in the methods section, use current definitions for sepsis, and if possible compare the mortality from their study group with the  earlier available mortality data from the previous study PMID: 39877396

Please submit your revised manuscript by May 05 2026 11:59PM. If you will need more time than this to complete your revisions, please reply to this message or contact the journal office at plosone@plos.org. When you're ready to submit your revision, log on to https://www.editorialmanager.com/pone/ and select the 'Submissions Needing Revision' folder to locate your manuscript file.

Please include the following items when submitting your revised manuscript:

- A letter that responds to each point raised by the academic editor and reviewer(s). You should upload this letter as a separate file labeled 'Response to Reviewers'.
- A marked-up copy of your manuscript that highlights changes made to the original version. You should upload this as a separate file labeled 'Revised Manuscript with Track Changes'.
- An unmarked version of your revised paper without tracked changes. You should upload this as a separate file labeled 'Manuscript'.

If you would like to make changes to your financial disclosure, please include your updated statement in your cover letter. Guidelines for resubmitting your figure files are available below the reviewer comments at the end of this letter.

If applicable, we recommend that you deposit your laboratory protocols in protocols.io to enhance the reproducibility of your results. Protocols.io assigns your protocol its own identifier (DOI) so that it can be cited independently in the future. For instructions see: https://journals.plos.org/plosone/s/submission-guidelines#loc-laboratory-protocols. Additionally, PLOS ONE offers an option for publishing peer-reviewed Lab Protocol articles, which describe protocols hosted on protocols.io. Read more information on sharing protocols at https://plos.org/protocols?utm_medium=editorial-email&utm_source=authorletters&utm_campaign=protocols.

We look forward to receiving your revised manuscript.

Kind regards,

Ramya Iyadurai

Academic Editor

PLOS One

Journal Requirements:

When submitting your revision, we need you to address these additional requirements.

1. Please ensure that your manuscript meets PLOS ONE's style requirements, including those for file naming. The PLOS ONE style templates can be found at

https://journals.plos.org/plosone/s/file?id=wjVg/PLOSOne_formatting_sample_main_body.pdf and

https://journals.plos.org/plosone/s/file?id=ba62/PLOSOne_formatting_sample_title_authors_affiliations.pdf

2. If the reviewer comments include a recommendation to cite specific previously published works, please review and evaluate these publications to determine whether they are relevant and should be cited. There is no requirement to cite these works unless the editor has indicated otherwise.

[Note: HTML markup is below. Please do not edit.]

Reviewers' comments:

Reviewer's Responses to Questions

**Comments to the Author**

1. Is the manuscript technically sound, and do the data support the conclusions?

The manuscript must describe a technically sound piece of scientific research with data that supports the conclusions. Experiments must have been conducted rigorously, with appropriate controls, replication, and sample sizes. The conclusions must be drawn appropriately based on the data presented.

Reviewer #1: Yes

Reviewer #2: No

Reviewer #3: Partly

Reviewer #4: Yes

**********

2. Has the statistical analysis been performed appropriately and rigorously?

Reviewer #1: I Don't Know

Reviewer #2: No

Reviewer #3: Yes

Reviewer #4: Yes

**********

3. Have the authors made all data underlying the findings in their manuscript fully available?

The PLOS Data policy requires authors to make all data underlying the findings described in their manuscript fully available without restriction, with rare exception (please refer to the Data Availability Statement in the manuscript PDF file). The data should be provided as part of the manuscript or its supporting information, or deposited to a public repository. For example, in addition to summary statistics, the data points behind means, medians and variance measures should be available. If there are restrictions on publicly sharing data—e.g. participant privacy or use of data from a third party—those must be specified.

Reviewer #1: Yes

Reviewer #2: No

Reviewer #3: Yes

Reviewer #4: Yes

**********

4. Is the manuscript presented in an intelligible fashion and written in standard English?

PLOS ONE does not copyedit accepted manuscripts, so the language in submitted articles must be clear, correct, and unambiguous. Any typographical or grammatical errors should be corrected at revision, so please note any specific errors here.

Reviewer #1: Yes

Reviewer #2: No

Reviewer #3: Yes

Reviewer #4: No

**********

5. Review Comments to the Author

Please use the space provided to explain your answers to the questions above. You may also include additional comments for the author, including concerns about dual publication, research ethics, or publication ethics. (Please upload your review as an attachment if it exceeds 20,000 characters)

Reviewer #1: Peer Review

Manuscript: PONE-D-26-06135

Prevalence of Sepsis and Outcome of Adult Patients Admitted to the Intensive Care Unit at a Tertiary Teaching Hospital in Tanzania: A Prospective Cohort Study

1. Summary

This manuscript reports a prospective observational study conducted in adult medical and surgical ICUs at Muhimbili National Hospital, Tanzania (May–November 2023). Among 248 ICU admissions, 103 patients met the authors' sepsis criteria, yielding a reported prevalence of 41.5% and an ICU mortality rate of 55.3%. The study further examines predictors of mortality, identifying higher SOFA scores, multiple comorbidities, and delayed antibiotic administration as key contributing factors.

The research topic is clinically significant given the substantial burden of sepsis in low- and middle-income countries (LMICs) and sub-Saharan Africa (SSA). However, major methodological and reporting concerns—particularly the sepsis case definition, internal inconsistencies in statistical methods, and high risk of time-related and severity confounding in the antibiotic timing analysis—currently preclude reliable interpretation of the findings.

2. Major Comments

1. Sepsis case definition is not aligned with Sepsis-3 and conflates screening tools with diagnostic criteria

The manuscript invokes the Sepsis-3 framework conceptually, yet the Methods section defines sepsis as qSOFA ≥ 2 and/or SIRS ≥ 2 and/or NEWS ≥ 5. These are screening or triage tools, not diagnostic definitions; their use is likely to produce substantial misclassification and poor comparability with the broader literature.

Required revision: Clearly distinguish between “suspected/possible sepsis” and “Sepsis-3–defined sepsis.” Where feasible, provide a sensitivity analysis using suspected infection plus SOFA increase ≥ 2 (or SOFA ≥ 2 within 24 hours), and report how prevalence estimates and effect measures change accordingly.

2. Study design is mislabeled as “cross-sectional” despite time-to-outcome follow-up

Patients were followed through ICU discharge or death, and the analysis examines longitudinal predictors of outcome—features consistent with a prospective cohort design rather than a true cross-sectional study.

Required revision: Revise the study design designation throughout the manuscript (title, Methods, and Abstract) and ensure that STROBE reporting elements align with cohort study standards.

3. Statistical methods are internally inconsistent and may be incorrectly implemented or interpreted

The Abstract and Results sections report modified Poisson regression with prevalence ratios (cPR and aPR), whereas the Methods section describes logistic regression. Furthermore, the multivariable results for SOFA score categories appear inconsistent with the observed crude mortality patterns—for example, a reported 100% mortality in the SOFA > 14 group is difficult to reconcile with adjusted PRs at or below 1.0 for certain categories.

Required revision: Align the statistical methods described in the Methods with those actually used in the Analysis. Provide a clear and internally consistent presentation of regression results, and verify that the direction and magnitude of adjusted estimates are analytically coherent.

3. Minor Comments

1. Provide a participant flow diagram illustrating the progression from total ICU admissions through exclusions and readmissions, to sepsis identified on admission versus ICU-acquired, and through to final outcomes.

2. Report missing data explicitly for key variables, including SOFA component scores, laboratory values, infection site determination, and antibiotic administration timing.

3. Clarify whether the “ICU-acquired infection” subgroup (onset ≥ 48 hours after admission) differs from early-onset cases with respect to causative pathogens and clinical outcomes. At minimum, present stratified descriptive results for this subgroup.

4. Recommendation

Decision: Major Revision

This manuscript addresses an important public health question with valuable ICU-level data from Tanzania. Nonetheless, the current version contains fundamental definitional and analytic problems—particularly regarding the sepsis case definition, potential antibiotic timing bias, and internal statistical inconsistencies—that must be resolved before the results can be interpreted with confidence. The authors are encouraged to address all major comments thoroughly and to revisit the study design classification, which appears inconsistent with the longitudinal nature of the data.

Reviewer #2: This manuscript addresses an important topic: the epidemiology and outcomes of sepsis in a tertiary ICU in a LMIC country. The study provides useful descriptive information on the burden of sepsis and mortality in this setting. However, several methodological and reporting issues limit the technical validity of the conclusions and require substantial revision.

1) the definition of sepsis is unclear and does not appear to follow the Sepsis-3 criteria. The manuscript states that sepsis was identified using qSOFA ≥2, SIRS ≥2, or NEWS ≥5. These scores are screening tools and should not be used as diagnostic criteria. The authors should clarify how sepsis was defined and consider aligning the case definition with Sepsis-3 (infection plus SOFA ≥2).

2) the analysis regarding “antibiotics within 1 hour” lacks a clearly defined time reference point (e.g., time of sepsis recognition, ED arrival, or ICU admission). Without a defined time zero, this variable is vulnerable to temporal bias and confounding by illness severity.

3) there is inconsistency in the statistical methods. The methods section describes logistic regression, while the results present modified Poisson regression estimates. The modeling strategy and rationale for the chosen regression approach should be clarified.

4) the multivariable model may be over-specified given the relatively small sample size and number of events. The authors should explain the variable selection process and consider simplifying the model.

Finally, the manuscript would benefit from language editing and improved table formatting. The data availability statement should also be clarified to ensure compliance with journal requirements.

Addressing these methodological and reporting issues would substantially improve the clarity and reliability of the findings.

Reviewer #3: Thank you for the opportunity to review “Prevalence of sepsis and outcome of adult patients admitted in an intensive care unit patients in a tertiary teaching hospital in Tanzania: A prospective cross-sectional Study”, which, as the title states, is a cross-sectional study of patients with sepsis at a single tertiary center in the nation of Tanzania. While the results of single-center cross sectional studies such as these are not usually practice-changing, they provide a profile of disease prevalence and characteristics in a particular geographic region which is relevant to the medical literature, particularly in lower and middle-income countries (LMICs) where resource availability may be different than that in the resource rich countries which produce most of the medical literature.

In terms of novelty, I do not find other papers addressing sepsis incidence and outcomes among adult ICU patients in the nation of Tanzania, though I did note this similar paper which examined sepsis outcomes among adult emergency department patients with sepsis in Tanzania (Bonnewell et al, Open Forum Infect Dis, 2024, PMID: 39877396).

Publication is possible, but significant changes to the analysis and conclusions would be needed.

Major concerns:

1. The criteria the authors have used to identify sepsis (qSOFA 2 or more, SIRS 2 or more, or NEWS 5 or more) are not entirely robust. Since the sepsis-3 criteria were published (Singer JAMA 2016), the use of qSOFA as a screening tool has fallen out of favor and is no longer recommended in the 2021 surviving sepsis guidelines. SIRS and NEWS may be used as screening tools, but an established diagnosis of sepsis is made by a SOFA score increase of 2 or more from baseline in concert with a known or suspected infection. The authors state that they calculated SOFA scores within 24 hours of ICU admission for all patients; using this as the basis for sepsis diagnoses would be more reliable than the screening tools, and results would be more readily comparable with results from other studies.

2. One of the authors’ main conclusions is that their observed ICU sepsis mortality rate of 55% is much higher than that observed in resource-rich countries. While resource availability is undoubtedly linked to survival rates, the degree of discrepancy should be further explored. I recommend one of two approaches. First, the authors could provide literature citations of mortality rates among ICU-admitted patients with sepsis in resource rich countries (the citations the authors provide all seem to apply to LMICs). Alternatively, the authors could state the number of septic patients in their study who also qualify as having septic shock (Shankar-Hari, JAMA, 2016, PMID: 26903336). This would give a better idea of how sick the cohort is and whether the expected mortality rate would be closer to the 10% seen in sepsis alone (Singer, JAMA, 2016) or the 42% expected for septic shock (Shankar-Hari).

3. The motivation for and application of the univariate regression analysis needs to be clearer. Use of the multivariable logistic regression analysis makes sense, but what was the univariable regression meant to show that couldn’t be determined from the other single variable analyses utilized by the authors (chi-squared and fisher exact testing)? It adds clutter to the results without significantly changing the conclusions; I would recommend removing the univariable regression analysis unless there was a specific purpose for which the authors have included it (if so, this should be stated).

4. The authors conclude that younger age is associated with improved survival because of a statistically significant decrease in sepsis mortality in the 26-30 year old age bracket, and such a conclusion would align with widely accepted prior research. However, the distribution of mortality risk, when taken as a whole, actually shows a relatively flat risk of sepsis mortality relative to age, particularly when adjusted odds ratios are considered (table 4). That the adjusted odds ratio of mortality for the 18-25 age group is identical to the >50 age group is particularly striking. Rather than trying to sweep over this the authors should lean into it and further explore in their discussion what could be driving it. To me this is the most interesting finding of the paper.

Minor concerns:

Introduction:

1. In the second paragraph the authors note several statistics as percentages but it’s unclear what the percentages refer to. “…with approximately 85% of all cases and 84% of all sepsis-related deaths”: is this saying 85% of all ICU admissions are patients with sepsis and 84% of all hospital deaths are sepsis related? This needs to be more clear. Similarly “…17% of all adult hospital admissions and 31% of ICU admissions” what are the 17% and 31% referring to?

2. In the third paragraph of the introduction (“Patients with sepsis…”), the authors should state the specific population they are referring to here. Are these statements about sepsis trends worldwide? Sepsis trends specific to LMICs? Or specific to a particular geographic region? Based on the citations used it appears to be specific to LMICs or particular parts of Africa, but that should be stated in the text.

3. In the fourth paragraph of the introduction, what are “under-fives”? This needs to be defined in the text.

Methods:

1. In study duration, I recommend writing out the dates of the study longhand (e.g. May 16, 2023) to avoid international differences related to ordering day and month in shortform.

2. Per the methods section, the MICU/SICU at the study site admits about 300 patients per year, but in 6 months of sampling there were 248 patients admitted and included in the study (suggesting a typical annual volume of more like 500 patients per year). There needs to be some explanation about how these numbers are coherent with one another.

3. More description of the capabilities of the study center would be helpful. Is continuous renal replacement therapy available? What about hemodialysis more generally? VA- or VV-ECMO? For subjects with severe sepsis-induced ARDS is there a protocol for prone positioning? Inhaled prostacyclins (e.g. velitri or flolan)?

4. An INR of >1.5 was utilized to establish a diagnosis of liver dysfunction, but among septic patients and elevated INR could also be caused by disseminated intravascular coagulopathy (DIC) or simply sepsis-associated coagulopathy.

5. The authors should state why in their singe variable analysis they sometimes used chi-squared testing and sometimes used Fisher Exact testing. It appears they are appropriately using Fisher Exact testing for variables with low frequency outcomes, but this should be explicitly stated.

Results:

1. In Table 3, p-values should not be expressed as “0”, but rather as an upper boundary (e.g. “P < 0.0001”).

2. [Related to major concern 3 above]: Table 4 is not very clear. What do the acronyms cPR and aPR refer to? I think these are some type of crude and adjusted odds ratios from the regression analyses but the acronyms should be defined. Additionally, the cPR and aPR column titles should be changed to match the format in which they are reported in the table (ie. “cPR (95% CI)”). Finally, if the univariate regression is kept (see major concern 3) the authors should be very deliberate about which regression analysis they are referring to. In the paragraph starting with “In a univariate modified Poisson analysis…” it appears both cPRs and aPRs are referenced without clear distinction between univariate and multivariate analysis models.

Discussion:

1. The authors note that their LOS among non-survivors was longer than that for non-survivors elsewhere in Sub Saharan Africa (SSA) and attribute this a slow decline after an initial period of stabilization. The important question raised by this, though, is why the patients in the study center were able to achieve the initial period of stabilization whereas this was not achieved at other sites in SSA. Does this have to do with the way resources are allocated, or something else? The authors should comment on this.

Typos:

Overall the manuscript reads well but the authors should again proofread their manuscript for typos. A few that I found:

1. The word "patient" appears duplicated in the title. I recommend modification to "Prevalence of Sepsis and Outcome among Adult Intensive Care Unit Patients in a Tertiary Teaching Hospital in Tanzania: A Prospective Cross-Sectional Study"

2. In the abstract background paragraph, I believe the authors intend "...particularly in low- and middle-income countries, which face a disproportionate burden...”

3. Last sentence of the abstract results paragraph should read "were independent predictors of high mortality"

4. At the top of the page containing Table 4, I believe the authors intend “were independent predictors of high mortality, and early initiation of antibiotics…”

5. In the discussion section titled Outcomes among adult patients with sepsis in the ICU, first paragraph, I believe the authors intend: “Early initiation of antibiotics allows rapid elimination of…”

Reviewer #4: The working group of Jongo et al. presents here data from original research in a single centre tertiary teaching hospital of Muhimbili University in Tanzania, it is a prospective Analysis of sepsis and the corresponding mortality rate in ICUs of this hospital.

2. Results reported have not been published elsewhere.

The results are not published elsewhere so far as we can evaluate this.

3. Experiments, statistics, and other analyses are performed to a high technical standard and are described in sufficient detail.

The scientific data is collected of 248 patients, inclusion criteria are mentioned, different scoring systems are mentioned. Was SOFA and News Score used the same way, in different patients. What is the exact sepsis definition they used? What was the exact criteria for diagnosing Coagulopathy/disseminated intravascular coagulation (DIC) to account for organ failure, was thrombocytopenia significant for diagnosing DIC?

4. Conclusions are presented in an appropriate fashion and are supported by the data.

The conclusions are presented appropriate, the comparison to exact data of other countries is missing, and the critical view on definitions of sepsis in other countries, i.e. criteria for diagnosing sepsis in DRG related countries.

It would be more elegant and interesting for the reader to place greater emphasis on the importance of early antibiotic administration, which is crucial for survival, and on the challenges of rapid yet accurate diagnosis through the early collection of blood cultures and antibiotic administration within one hour. This fact also presents a significant challenge for Western countries; the authors' perspective on this would be interesting.

5. The article is presented in an intelligible fashion and is written in standard English.

In the part of restriction, I don’t understand “6 period time and asses to care provisions “, please explain on this.

6. The research meets all applicable standards for the ethics of experimentation and research integrity.

The research integrity and standards is adherent to reporting guideline and community standard.

All in all, the paper is well written, data should be more focused on precise sepsis definition, this is even difficult for other countries, what is the problem in Tanzania, it is lacking a critical view on documentation problems, that is normal for this diagnosis.

7. The article adheres to appropriate reporting guidelines and community standards for data availability.

The article adheres to the standard guidelines of scientific writing; I miss a critical examination of the conditions that lead to the diagnosis of sepsis or sepsis with organ complications.

**********

6. PLOS authors have the option to publish the peer review history of their article (what does this mean?). If published, this will include your full peer review and any attached files.

If you choose “no”, your identity will remain anonymous but your review may still be made public.

**Do you want your identity to be public for this peer review?**  For information about this choice, including consent withdrawal, please see our Privacy Policy.

Reviewer #1: No

Reviewer #2: No

Reviewer #3: **Yes:** David A Wacker

Reviewer #4: No

**********

[NOTE: If reviewer comments were submitted as an attachment file, they will be attached to this email and accessible via the submission site. Please log into your account, locate the manuscript record, and check for the action link "View Attachments". If this link does not appear, there are no attachment files.]

To ensure your figures meet our technical requirements, please review our figure guidelines: https://journals.plos.org/plosone/s/figures

You may also use PLOS’s free figure tool, NAAS, to help you prepare publication quality figures: https://journals.plos.org/plosone/s/figures#loc-tools-for-figure-preparation.

NAAS will assess whether your figures meet our technical requirements by comparing each figure against our figure specifications.

Attachment Submitted filename: Review for manuscript PONE-D-26-06135.docx

Attachment Submitted filename: sepsis in tanzania review.docx

# Author comment: Author response to Decision Letter 1

**DOI:** 10.1371/journal.pone.0351137.r002

4 May 2026

Response to Reviewers

Manuscript ID: PONE-D-26-06135

Title: Prevalence, Outcomes, and Predictors of Mortality Among Adult Intensive Care Unit Patients with Sepsis at a Tertiary Hospital in Tanzania: A Prospective Cohort Study

Dear Editor and Reviewers,

We sincerely thank you for your thorough and insightful review of our manuscript. We appreciate the constructive comments, which have significantly improved the clarity, methodological rigor, and overall quality of our work. We have carefully addressed all comments and revised the manuscript accordingly. Below is a detailed, point-by-point response.

REVIEWER 1 – MAJOR COMMENTS

1. Sepsis definition not aligned with Sepsis-3

Response:

We agree with this important observation. The manuscript has been revised to clearly distinguish between screening and diagnostic criteria. All cases were included in the study after evidence of infection. This was included in the previous submission on the description of sources of infection.

Where feasible, we have clarified the implications of this definition and improved comparability with existing literature.

2. Study design mislabeled as cross-sectional

Response:

We agree and have corrected this throughout the manuscript. The study is now consistently described as a prospective cohort study, as patients were followed from ICU admission until discharge or death. The title, abstract, and methods have been revised accordingly.

3. Statistical inconsistencies (Poisson vs logistic regression, SOFA findings)

Response:

We thank the reviewer for highlighting this inconsistency. The statistical methods have been fully revised for clarity and consistency. The manuscript now exclusively reports modified Poisson regression for estimation of adjusted prevalence ratios (aPR). The regression models have been re-evaluated, and SOFA categories were restructured to ensure logical consistency with observed mortality patterns.

REVIEWER 2 – MAJOR COMMENTS

1. Sepsis criteria robustness

Response:

We have revised the sepsis definition to align with Sepsis-3 criteria and clarified the role of screening tools. This improves diagnostic accuracy and comparability with global literature.

2. High mortality compared to resource-rich settings

Response:

We have added the literature on the HIC as advised.

3. Univariate regression analysis redundancy

Response:

In the revised analysis, we have removed the Chi-square and Fisher's exact testing and left the univariate analysis, which provided the strength and level of significance. Those variables with a p-value < 0.2 were included in the multivariable analysis regression model to control for confounding.

4. Age and mortality interpretation

Response:

We appreciate this insightful observation. We have revised the interpretation to reflect that mortality risk was relatively uniform across age groups after adjustment.

Although one subgroup (26–30 years) showed statistical significance, this was not consistent across other groups, and confidence intervals were wide. We now interpret this finding as suggesting that illness severity, rather than age alone, is the dominant determinant of outcome.

MINOR COMMENTS

1. Introduction, unclear percentages

Response:

We have revised the text to explicitly define all percentages. The denominators (global cases, global deaths, hospital admissions, ICU admissions) are now clearly specified.

2. Population clarification (LMIC vs global vs regional)

Response:

We have clarified that the referenced trends relate specifically to LMICs and sub-Saharan Africa.

3. Definition of “under-fives.”

Response:

The exact term and age group studied have been revised.

4. Study duration format

Response:

Dates are now written in full format (e.g., May 16, 2023).

5. ICU admission volume discrepancy

Response:

We have clarified that increased admissions during the study period likely reflect referral patterns and variability in patient flow.

6. ICU capability description

Response:

We have expanded the Methods section to describe ICU resources, including availability of mechanical ventilation, vasopressors, intermittent hemodialysis, CRRT, and absence of ECMO.

7. INR and liver dysfunction (DIC concern)

Response:

We revised the definition of acute liver injury to avoid reliance on INR alone and clarified that coagulopathy in sepsis may reflect DIC rather than hepatic dysfunction.

8. P-values reported as 0

Response:

All such values have been corrected to “p < 0.001”.

9. Table clarity (cPR/aPR definitions)

Response:

The cPR and aPR acronyms have been defined. An appropriate description of cPR and aPR has been revised in the results section.

10. ICU length of stay explanation.

Response:

We have expanded the discussion to explain longer LOS among non-survivors in our setting, highlighting differences in early stabilization, resource availability, and disease trajectory.

11. Typographical errors

Response:

All typographical and grammatical issues have been corrected as suggested.

ADDITIONAL REVISIONS (EDITOR COMMENTS)

Participant flow diagram

Response:

A flow diagram has been added showing ICU admissions, sepsis identification, subgroup classification, and outcomes.

Missing data reporting

Response:

In our study, we had 3 patients whose source of infections undocumented, which we labeled as undetermined in the source of infection in Table 1

ICU-acquired vs early-onset sepsis

Response:

We have added a stratified descriptive comparison of early-onset and ICU-acquired sepsis, including clinical characteristics and outcomes.

CONCLUSION

We believe that these revisions have addressed all major and minor concerns raised by the reviewers and have substantially strengthened the manuscript. We are grateful for the constructive feedback and hope the revised version meets the journal’s standards for publication.

Sincerely,

Atala Jongo

On behalf of all authors

Attachment Submitted filename: Response to Reviewers.docx

# Editor report: Decision Letter 1

**DOI:** 10.1371/journal.pone.0351137.r003

**Authors:** Ramya Iyadurai

25 May 2026

<p>Prevalence, Outcomes, and Predictors of Mortality Among Adult Intensive Care Unit Patients with Sepsis at a Tertiary Hospital in Tanzania: A Prospective Cohort Study

PONE-D-26-06135R1

Dear Dr. JONGO,

We’re pleased to inform you that your manuscript has been judged scientifically suitable for publication and will be formally accepted for publication once it meets all outstanding technical requirements.

Within one week, you’ll receive an e-mail detailing the required amendments. When these have been addressed, you’ll receive a formal acceptance letter and your manuscript will be scheduled for publication.

An invoice will be generated when your article is formally accepted. Please note, if your institution has a publishing partnership with PLOS and your article meets the relevant criteria, all or part of your publication costs will be covered. Please make sure your user information is up-to-date by logging into Editorial Manager at Editorial Manager® and clicking the ‘Update My Information' link at the top of the page. For questions related to billing, please contact billing support.

If your institution or institutions have a press office, please notify them about your upcoming paper to help maximize its impact. If they’ll be preparing press materials, please inform our press team as soon as possible -- no later than 48 hours after receiving the formal acceptance. Your manuscript will remain under strict press embargo until 2 pm Eastern Time on the date of publication. For more information, please contact onepress@plos.org.

Kind regards,

Ramya Iyadurai

Academic Editor

PLOS One

Additional Editor Comments (optional):

None

Reviewers' comments:

# Editor report: Acceptance letter

**DOI:** 10.1371/journal.pone.0351137.r004

**Authors:** Ramya Iyadurai

PONE-D-26-06135R1

PLOS One

Dear Dr. JONGO,

I'm pleased to inform you that your manuscript has been deemed suitable for publication in PLOS One. Congratulations! Your manuscript is now being handed over to our production team.

At this stage, our production department will prepare your paper for publication. This includes ensuring the following:

* All references, tables, and figures are properly cited

* All relevant supporting information is included in the manuscript submission,

* There are no issues that prevent the paper from being properly typeset

You will receive further instructions from the production team, including instructions on how to review your proof when it is ready. Please keep in mind that we are working through a large volume of accepted articles, so please give us a few days to review your paper and let you know the next and final steps.

Lastly, if your institution or institutions have a press office, please let them know about your upcoming paper now to help maximize its impact. If they'll be preparing press materials, please inform our press team within the next 48 hours. Your manuscript will remain under strict press embargo until 2 pm Eastern Time on the date of publication. For more information, please contact onepress@plos.org.

You will receive an invoice from PLOS for your publication fee after your manuscript has reached the completed accept phase. If you receive an email requesting payment before acceptance or for any other service, this may be a phishing scheme. Learn how to identify phishing emails and protect your accounts at https://explore.plos.org/phishing.

If we can help with anything else, please email us at customercare@plos.org.

Thank you for submitting your work to PLOS ONE and supporting open access.

Kind regards,

PLOS ONE Editorial Office Staff

on behalf of

Dr. Ramya Iyadurai

Academic Editor

PLOS One
